# Comparative genomic analysis of Escherichia coli isolated from cases of bovine clinical mastitis and the dairy farm environment

**DOI:** 10.1099/mgen.0.001436

**Published:** 2025-06-24

**Authors:** Dongyun Jung, Soyoun Park, Janina Ruffini, Forest Dussault, Simon Dufour, Jennifer Ronholm

**Affiliations:** 1 Faculty of Agricultural and Environmental Sciences, Macdonald Campus, McGill University, Ste Anne de Bellevue, Québec, Canada; 2 Regroupement FRQNT Op+Lait, Saint-Hyacinthe, Québec, J2S 2M2, Canada; 3 Mastitis Network, Saint-Hyacinthe, Québec, J2S 2M2, Canada; 4 Health Canada, Ottawa, Ontario, Canada; 5 Faculté de médecine vétérinaire, Université de Montréal, Saint-Hyacinthe, Québec, Canada

**Keywords:** comparative genomics, environmental bovine mastitis, mammary pathogenic *Escherichia coli*, whole-genome sequencing

## Abstract

*Escherichia coli* is a major causative agent of environmental bovine mastitis, and this disease causes significant economic losses for the dairy industry. There is still debate in the literature as to whether mammary pathogenic *E. coli* (MPEC) is indeed a unique *E. coli* pathotype or if this infection is merely an opportunistic infection caused by any *E. coli* isolate being displaced from the bovine gastrointestinal tract to the environment and then into the udder. In this study, we conducted a thorough genomic analysis of 113 MPEC isolates from clinical mastitis cases and 100 environmental *E. coli* isolates from the environment of dairy farms around the world. A phylogenomic analysis indicated that MPEC and the environmental *E. coli* isolates formed clades based on common sequence types and O antigens but did not cluster based on mammary pathogenicity. The comparison of core and soft-core genes of each set of isolates identified the three genes of the ferric dicitrate uptake system (Fec), *fecI*, *fecR* and *fecA*, as soft-core genes of MPEC (*n*=110). These genes were also present in 27 *E. coli* isolates from environmental sources. Rather than being a virulence gene cluster, it is likely that the Fec system provides a competitive advantage to *E. coli* in the mammary gland – an iron-poor environment. Using this cluster as a marker for MPEC may offer an opportunity to develop novel treatments for *E. coli* mastitis, based on its presence.

## Data Summary

Sequencing data and genome assemblies are available at DDBJ/ENA/GenBank as BioProject PRJNA612640 under the accession numbers JAASLI000000000–JAASQG000000000. All supporting data and protocols have been provided within the article and supplementary data files.

**Impact Statement**

Mammary pathogenic *Escherichia coli* (MPEC) is a common cause of mastitis in dairy cattle. It is still controversial as to whether MPEC is a unique *E. coli* pathotype since a core set of virulence factors that are unique to MPEC has not yet been defined. Our comparative genomics analysis of MPEC and environmental *E. coli*, isolated from dairy farms, identified the *fec* operon as being significantly more common in MPEC than in environmental isolates, and we were unable to identify a set of virulence genes that are unique to MPEC. The *fec* operon encodes genes involved in iron acquisition which likely confer a competitive advantage to *E. coli* in the mammary gland. Given the high concentrations of ferric citrate in bovine milk, the Fec system may be a necessary factor for MPEC to survive in the bovine udder where the abundance of iron is severely restricted. Further highlighting the critical nature of the *fec* operon to *E. coli* mastitis indicates that this system may be a good target for future MPEC diagnostics and treatments.

## Introduction

Bovine mastitis – inflammation of bovine udder usually caused by a bacterial infection – is a costly disease in the dairy industry [1] and results in annual losses of $665 million (CAD) for the Canadian dairy industry [2], $2 billion (USD) for the American dairy industry [3] and £168 million for the British dairy industry [4]. The aetiological agents of bovine mastitis can be categorized as either contagious or environmental pathogens. Contagious bovine mastitis is commonly caused by *Staphylococcus aureus*, *Streptococcus agalactiae*, *Mycoplasma bovis* and *Corynebacterium bovis* which are transmitted from infected to uninfected cows via milking equipment, direct contact or vectors like farm workers. Modern dairy farm practices, including early mastitis prevention programmes, were focused on controlling contagious mastitis, and now, as a result, environmental mastitis is the most common form of this disease [5]. Environmental mastitis pathogens originate from the farm environment such as pasture, stable or bedding material. The bovine gastrointestinal (GI) tract is a common source for environmental pathogens [5]. *Escherichia coli* is a very common aetiological agent of environmental mastitis [6,7].

*E. coli* is a genetically and phenotypically diverse bacterial species. The range of *E. coli* diversity is particularly apparent in terms of host–bacteria relationships where it can be a mutualist, commensal, pathogen or occasional symbiont in the GI tract of a variety of host species [8]. Pathogenic strains are broadly categorized in humans and animals as either diarrheagenic *E. coli* or extraintestinal pathogenic *E. coli* (ExPEC). ExPEC typically resides asymptomatically within the intestine but causes severe infection when allowed to colonize extraintestinal niches [9]. Within each broad group, there are several sub-groups of strains that share virulence factors and similar clinical manifestations which are known as pathotypes [10]. Uropathogenic *E. coli* (UPEC), which is the etiological agent of about 90% of human urinary tract infections [11], has been relatively recently recognized as a distinct ExPEC pathotype [10]. This infection was once thought to be an opportunistic infection caused solely by displacement of any intestinal *E. coli* into the urinary tract [10], but now, it is known that only a distinct subset of *E. coli*, originating from the GI tract, results in UPEC infections [12]. Most virulent UPEC strains are from the B2 lineage among other main *E. coli* phylogroups A, B1 and D [13]. Many pathogenicity islands (PAIs) are associated with UPEC, and these islands can carry important virulence factors, specifically: P fimbriae, type I fimbriae, haemolysins, iron acquisition proteins, bacteriocins and the *malX* gene which is associated with the phosphotransferase system enzyme II that uses glucose and maltose as the main substrates [14,16].

The existence of a distinct mammary pathogenic *E. coli* (MPEC) pathotype has been proposed [9], but defining virulence factors of this group has not yet been identified [17]. The lack of a set of virulence genes common to all MPEC isolates, despite several attempts to identify them [17,21], has led to a proposed model for this disease where the mere introduction of any GI originating *E. coli* into the mammary gland and the resultant inflammatory response can result in clinical mastitis [22,23]. In this model, the severity of *E. coli* clinical mastitis is primarily dependent on host factors. However, this model fails to explain several aspects of *E. coli* clinical mastitis. For example, not all *E. coli* strains can cause clinical mastitis in experimental models of the disease [24], and mastitis strains are much less genetically diverse than bovine commensal *E. coli* [17,21].

The Fec system, ferric dicitrate uptake system, appears to be much more common in MPEC isolates than in other isolates derived from dairy cow environments. In addition, the Fec system is over-expressed when MPEC strains are grown in milk, and Fec knockouts are unable to induce clinical mastitis [25,26]. The presence of the Fec system may explain the adaptability of *E. coli* in the iron-poor environment of the bovine mammary gland [27]. However, the complex aetiology of *E. coli* adaptability and virulence is not fully understood yet.

In this study, we advance upon previous work by performing a detailed genomic analysis of 113 MPEC isolates. To identify the genetic traits that differentiate MPEC isolates from other bovine *E. coli* isolates, we performed a comparative genomic analysis in which MPEC isolates were compared to 100 environmental *E. coli* isolates from dairy cattle habitats that were not associated with the disease.

## Methods

### MPEC isolates and genomes of environmental *E. coli*

MPEC isolates (*n*=113) were obtained in 2019 from the Mastitis Pathogen Culture Collection which is maintained and curated by the Canadian Bovine Mastitis Research Network [28]. Each isolate was obtained from milk samples originating from 113 different cows from 57 herds (Alberta=9, Ontario=17, Quebec=17 and Atlantic provinces=14) experiencing clinical mastitis either on the day of diagnosis (*n*=100) or on subsequent post-clinical mastitis follow-up sampling (within 14 days, *n*=7; between 14 and 28 days, *n*=6) between 2007 and 2008 [28]. As previously described, MPEC isolates were isolated on a bi-plate containing Columbia agar with 5% sheep blood and MacConkey agar, and biochemical tests were performed to confirm that the isolates were *E. coli* (lactose and indole positive, oxidase and citrate negative) [29]. Bovine metadata, including herd number and location, cow ID, quarter position, sampling data, mastitis severity score [30], days in milk at sampling and cow’s parity, are summarized in Table S1, available in the online Supplementary Material.

The whole genomes of 100 bovine *E. coli* isolates, not associated with bovine disease, were obtained from the National Center for Biotechnology Information (NCBI) database. These genomes were derived from isolates from bovine faeces, skin, cowsheds and milking areas, as described in previous studies, and originated from a variety of international locations, excluding Canada [17,25, 31, 32] (Table S2). The environmental *E. coli* genomes were sequenced using different short-read sequencers, depending on the study, including the Illumina MiSeq, Illumina HiScanSQ, and 454 GS FLX Titanium, and assembled using different *de novo* assembly tools, including Platanus v1.2.2, Newbler v2.3 [31]. CLC Genomics Workbench v.6.5.2 [25] and SPAdes v3.1.1 and v3.5.0 [17,32] (Table S2).

### Whole-genome sequencing, assembly and annotation

Each MPEC isolate was streaked on tryptic soy agar (Becton Dickinson, NJ), and incubated overnight at 37 ℃. A single colony was picked and incubated in tryptic soy broth (Becton Dickinson, NJ) overnight at 37 ℃ at 200 r.p.m. DNA was extracted from each isolate with a culture that had >1×10^8^ cells ml^−1^ (OD_600nm_ > 0.8) at the time of extraction using DNAzol reagent (Invitrogen, CA) following the manufacturer’s instructions. DNA was further purified using the Qiagen DNeasy PowerClean Pro Cleanup Kit (QIAGEN, Hilden, Germany), as per the manufacturer’s instructions. DNA from isolates that did not produce high-quality DNA via this method was re-extracted using the Maxwell RSC and the recommended Blood DNA kit (Madison, WI, USA) according to the manufacturer’s instructions. A DNA concentration between 10 and 100 ng µl^−1^ with corresponding purity measurements of A260/280>1.8 and A260/230 between 1.8 and 2.2 based on NanoDrop measurements (Thermo Fisher, MA) was achieved prior to each sequencing library preparation.

DNA was further quantified using the Quant-iT dsDNA Assay kit prior to library preparation (Thermo Fisher, MA). DNA library preparation was performed using the Illumina Nextera DNA Flex Library Prep kit (Illumina, CA) optimized for short-read sequencing by the Illumina MiSeq system as per the manufacturer’s instructions. The tagmentation step was optimized to 15 minutes to achieve a DNA target length of 500–600 bp followed by a clean-up step. Tagmented DNA was amplified using Nextera DNA CD indexes via PCR followed by a clean-up step and concentration check. A pooled library was made combining all samples into one 1.5-ml tube, and a final quantification step was performed to ensure a final concentration of 1.6 ng µl^−1^ (4 nM). After library pool denaturation was performed by adding sodium hydroxide, a final concentration of 12 pM was obtained, and a PhiX control was added to a concentration of 20 pM. The library and PhiX control were loaded into a MiSeq v3 reagent kit, and 600 cycles (300 forward and 300 reverse) of sequencing were conducted using a MiSeq benchtop sequencer (Illumina).

Sequence reads were *de novo* assembled using the software pipeline ProkaryoteAssembly version 0.1.6 (https://github.com/bfssi-forest-dussault/ProkaryoteAssembly). This pipeline includes quality control and trimming of low-quality sequences (*Q* value<20) using BBDuk (BBMap v38.79), error-correction using Tadpole (BBMap), assembly using Skesa v2.4, alignment of the error-corrected reads against draft assembly BBMap and polishing of the assembly using Pilon v1.23 [33,35]. After assembly, contigs shorter than 1 kbp were discarded, and the coverage and contigs were quantified using Qualimap [36]. Prokka was used to annotate the assembled contigs of genomes of MPEC and environmental *E. coli* [37]. The pipeline includes annotation of protein-coding genes by identifying coordinates of candidate genes from ISfinder, UniProt, Pfam and TIGRFAMs [38,42].

### Pan-genome analysis

Roary v3.13 was used to construct a pan-genome for MPEC and the environmental *E. coli* isolates to allow for a direct comparison between the two groups of genomes. Paralog splitting options were not used [43]. Roary was run using the command line, roary -e *gff. The predicted functional proteins coded in the pan-genome of MPEC and environmental *E. coli* sets were identified by clustering orthologous groups (COG) on eggNOG-mapper using the representative nucleotide sequences from each of the clusters in the pan-genomes (*E*-value <1×10^−10^) [44,45]. The core genome alignment file from Roary was used as input for IQ-TREE which uses the ModelFinder Plus algorithm, selects the best-performing substitution model and builds a phylogenomic tree with it [46,47]. Specifically, the GTR+F+R10 model was used on IQ-TREE to build the phylogenomic tree of MPEC and environmental *E. coli* genomes. To visualize the tree, Interactive Tree Of Life (iTOL) v4 (https://itol.embl.de) was used [48].

The lists of genes from MPEC and environmental *E. coli* isolates were retrieved from the gene_presence_absence.csv file generated by Roary. The gene-by-gene pairwise comparison was done between core and soft-core genes of MPEC and all the genes of the environmental *E. coli* isolates using Venny v.2.1 [49]. Scoary v1.6.12 was also used to determine the differences in the prevalence of the genes between MPEC and environmental *E. coli* isolates [50]. The core genes of MPEC that are not present in environmental *E. coli* isolates identified by both Venny and Scoary were classified as unique genes in MPEC. The presence of any common or unique genes in both genome sets was confirmed by manual screening on individual genomes using nucleotide sequences of the common or unique genes by standalone BLAST +v2.12.0 [51].

### Identification of sequence type, O and H antigens, plasmid replicon types and genomic islands

Sequence types (STs) of each isolate were identified using the tool mlst (https://github.com/tseemann/mlst) which incorporates data from the PubMLST database [52]. To identify the distribution of O and H serotypes, ABRicate v1.0 (https://github.com/tseemann/abricate) was used with the EcOH database for O and H serotypes [53]. Minimum coverage and identity settings for the screening were set to 90%. ABRicate was used to identify plasmid replicon types, using the PlasmidFinder v2.1 database and plasmid multi-locus sequence typing (pMLST v2.0) was performed on the most prevalent replicon types in MPEC and environmental *E. coli* genomes (https://github.com/genomicepidemiology/plasmidfinder) [54]. We investigated the evidence of horizontal transfer of potential marker genes of MPEC by predicting genomic islands (GIs). Putative GIs were predicted using IslandViewer 4 using IslandPath-DIMOB and SIGI-HMM as island prediction methods. The previously closed *E. coli* ECC-1470 genome was used as a reference strain (accession number: NZ_CP010344.1) (>8 kbp as cut-off) [55]. The identified predicted GIs were screened to see if any unique genes of MPEC were present.

## Results

### Quality of sequenced genomes of MPEC and environmental *E. coli*

The assembly of each draft genome for the MPEC isolate was evaluated, and coverage and number of contigs are reported in Table S1. The range of coverage for individual genomes was between 22X and 360X, and the number of contigs ranged from 28 to 149. The genomes of environmental *E. coli* were selected from those available in the NCBI database based on isolation from dairy cattle environments, including cowsheds, faeces, skin and gastrointestinal tracts, or from the milking room, having coverage between 20X and 90X and having less than 419 contigs [17,18, 25, 31, 32, 56, 57].

### Absence of major clusters of MPEC by origin, herds and provinces

**Fig. 1.**
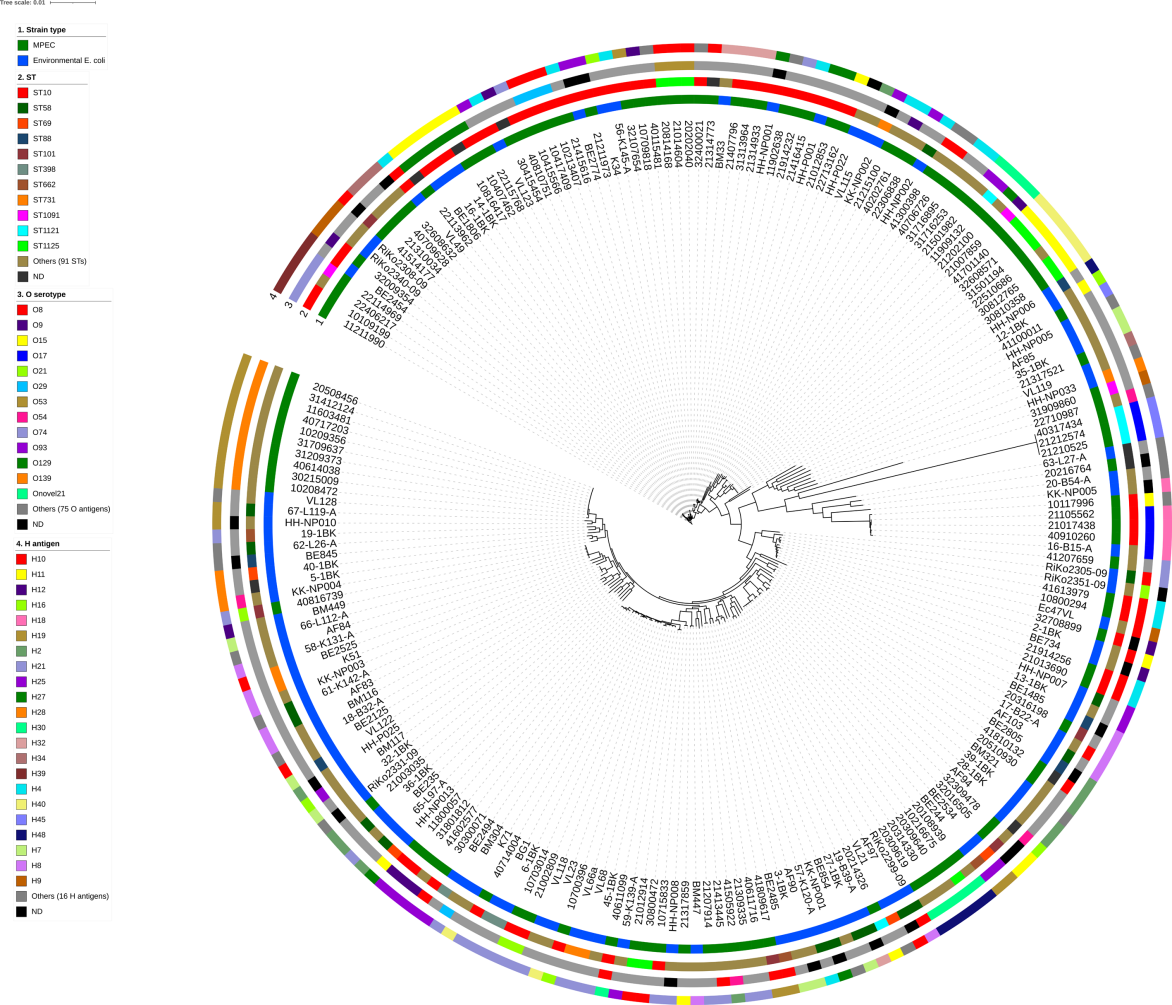
Phylogenomic tree of clinical mastitis-related MPEC and environmental *E. coli* isolates by cgSNPs. A phylogenomic tree was constructed using IQ-TREE based on core genomes of MPEC and the environmental *E. coli* genomes. The tree was visualized using iTOL v4, and each genome was annotated with sequence types by MLST (*n*=103), O (*n*=88) and H-antigens (*n*=38).

A phylogenomic tree which illustrates the relatedness of the 113 MPEC and 100 environmental *E. coli* isolates examined in this study was created by comparing core genome single nucleotide polymorphisms (cgSNPs) (Fig. 1). There was no apparent clustering of MPEC or environmental *E. coli* isolates based on origin, herds or provinces, and MPEC isolates were not phylogenetically differentiated from environmental *E. coli* isolates. There was a large range in the diversity of isolates in this study, which included 103 different STs, 88 different O-antigens and 38 different H-antigens. STs, O and H antigens of each genome are indicated in Tables S1 and S2. The most common STs identified in this study were ST10 and ST58.

### Comparative genomic analysis between MPEC and environmental *E. coli* isolates

The pan-genomes of MPEC and the environmental *E. coli* isolates were constructed using Roary after assembly and annotation of each genome. A total of 25,228 gene clusters were grouped from 213 *E. coli* genomes. Total genes consist of 3,420 core and soft-core genes (shared by 99–100% of genomes and 95–99% of genomes, respectively), 1,605 shell genes (shared by 15–95% of included genomes) and 20,203 cloud genes (shared by 0–15% of genomes).

**Fig. 2.**
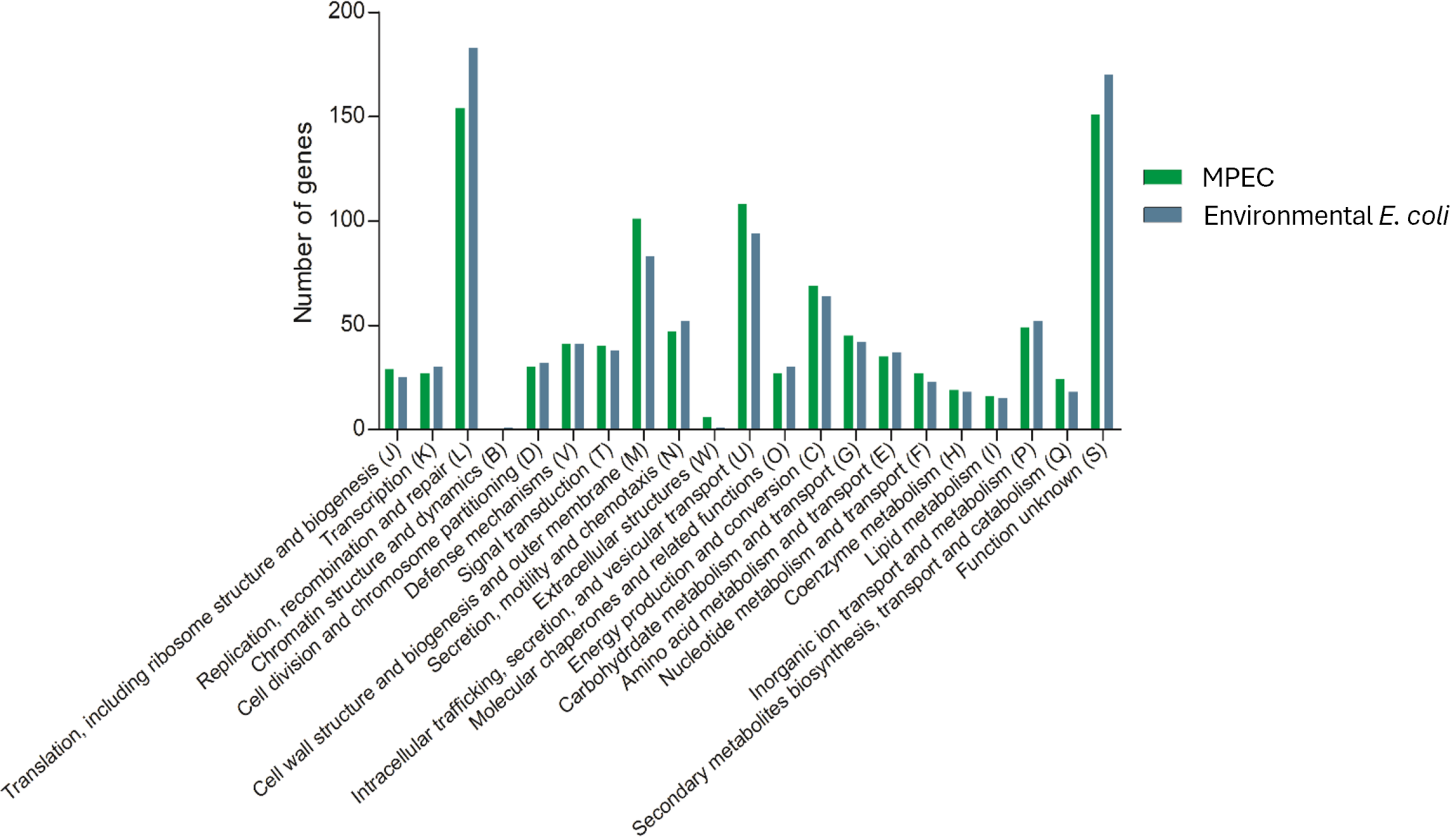
COGs of pan-genes of clinical mastitis-related MPEC and environmental *E. coli*. The groups were identified using eggNOG-mapper with *E*-value <1×10^−10^. The COGs are related to information storage and processing (groups J, K, L), cellular processes and signalling (groups D, V, T, M, N, W, U, O), metabolism (groups C, G, E, F, H, I, P, Q) and uncharacterized functions (group S).

**Fig. 3.**
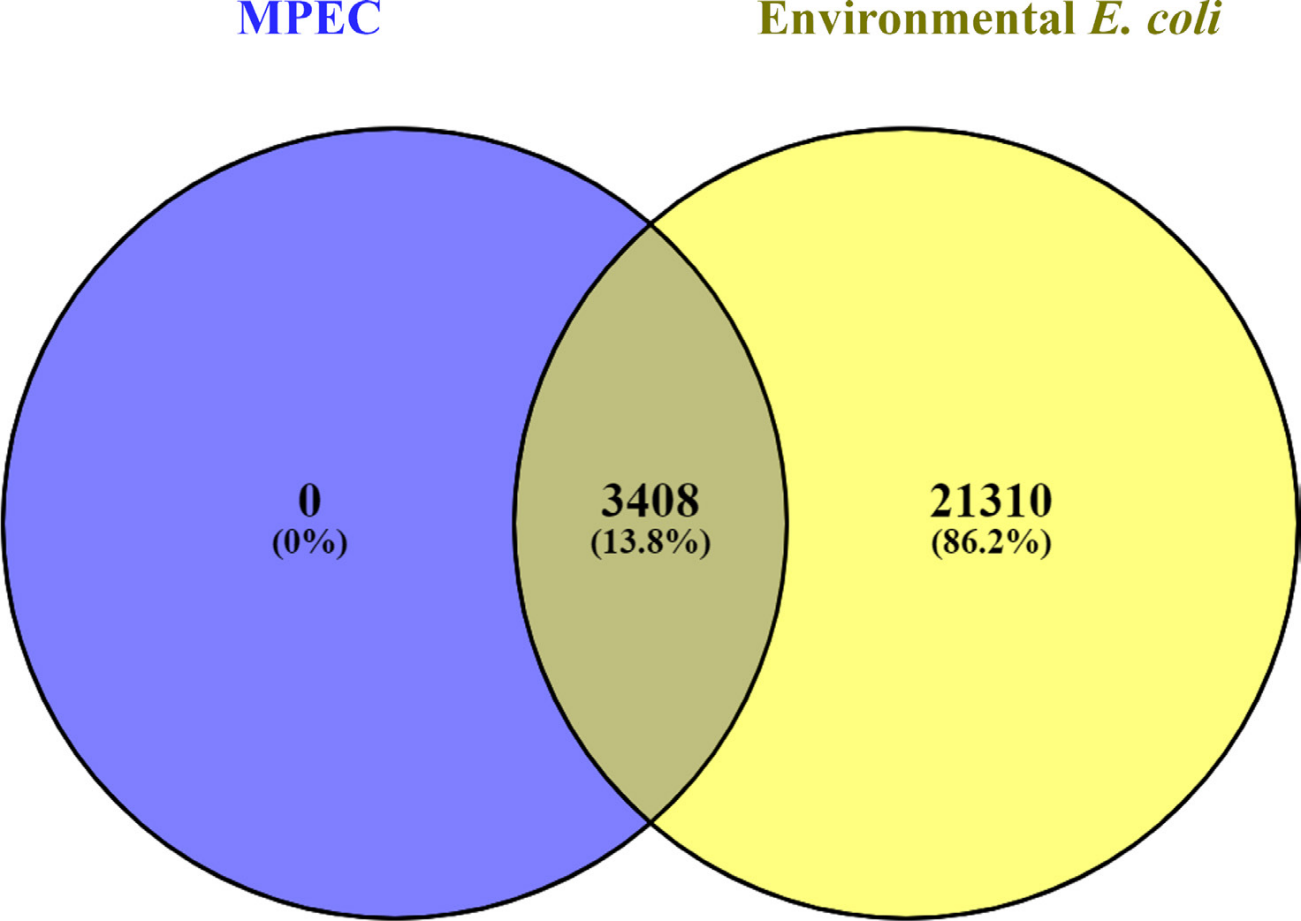
The number of core and soft-core genes in clinical mastitis-related MPEC and all genes in environmental *E. coli* genomes in the Venn diagram. The names of all genes of each genome set were excerpted from the pan-genome result by Roary.

The pan-genome of MPEC and the environmental *E. coli* genomes were compared via functional classification by COGs. There was no significant difference between COGs of MPEC and the environmental *E. coli* genomes (*P*=0.85) (Fig. 2). To identify core and soft-core genes that are unique to MPEC relative to the other bovine-associated isolates, a gene-by-gene pairwise comparison between core and soft-core genes of MPEC and all genes of the environmental *E. coli* isolates was performed using Venny (Fig. 3). All the core and soft-core genes were present in environmental *E. coli* genomes, indicating the absence of unique genes in MPEC.

The differences in the prevalence of genes in MPEC and the environmental *E. coli* genomes were identified by Scoary. The *fec* operon genes were core or soft-core genes in MPEC and statistically significantly associated with MPEC, while they were shell genes in the environmental *E. coli* genomes (Benjamini–Hochberg adjusted *P*<0.05); *fecI, fecR* and *fecA* were soft-core genes in MPEC genomes (*n*=110) and were present in 27 environmental *E. coli* genomes. The other *fec* operon genes, *fecC* and *fecB*, *fecD* and *fecE*, were soft-core and shell genes in MPEC genomes, respectively (*fecC*; *n*=108, *fecB,D,E*; *n*=106), and were present in 26 environmental *E. coli* genomes (Table 1).

**Table 1.** Prevalence of *fec* operon genes in clinical mastitis-related MPEC and environmental *E. coli* genomes

Gene	Putative annotation	Relative abundance in MPEC isolates (*n*/113)	Relative abundance in environmental *E. coli* isolates (*n*/100)
*fecI*	Putative RNA polymerase sigma factor	110/113	27/100
*fecR*	Regulator of iron dicitrate transporter	110/113	27/100
*fecA*	Fe(^3+^) dicitrate transport protein	110/113	27/100
*fecB*	Fe(^3+^) dicitrate-binding periplasmic protein	106/113	26/100
*fecC*	Fe(^3+^) dicitrate transport system permease protein	108/113	26/100
*fecD*	Fe(^3+^) dicitrate transport system permease protein	106/113	26/100
*fecE*	Fe(^3+^) dicitrate transport ATP-binding protein	106/113	26/100

### Analysis of mobile genetic elements in MPEC

The replicon types of plasmids in MPEC and the environmental *E. coli* genomes were screened using ABRicate (https://github.com/tseemann/abricate) based on the PlasmidFinder database [54]. In the MPEC isolates from this study, 25 plasmid replicon types were identified, and 31 plasmid replicon types were identified in the environmental *E. coli* isolates (Tables S1, S2 and S3). The plasmid IncF was the most prevalent replicon type in both MPEC (*n*=79) and the environmental *E. coli* (*n*=76) isolates. IncF replicon sequence typing was conducted to identify the difference between IncF-type plasmid replicons in 79 MPEC and 76 environmental *E. coli* genomes. The most prevalent replicon of IncF type plasmid was IncFIB (AP001918), which was identified in 61 out of 79 MPEC genomes and 67 out of 76 environmental *E. coli* genomes (Table S4). One novel allele of each of FIA and FII was found in 20 MPEC isolates, while this novel allele was only found in one environmental *E. coli* isolate (Table S5). Nineteen of the 20 MPEC genomes with two novel alleles of FIA and FII contained replicons of IncFIB (AP001918) and IncFIC (FII).

IslandPath-DIMOB and SIGI-HMM prediction methods were used within the IslandViewer 4 platform to predict the presence of GIs in MPEC genomes using an alignment-based strategy and the closed MPEC genome *E. coli* ECC-1470. Each MPEC isolate contained between 14 and 35 predicted genomic islands, but it was unclear if the *fec* operon was carried on a GI in most isolates.

## Discussion

In this study, 113 MPEC isolated from dairy cattle with clinical mastitis and 100 *E. coli* isolated from the environment on dairy farms were sequenced, and their genomes were analysed. Analysis indicated that these 213 strains were highly diverse and included 88 O-antigens and 38 H-antigens. However, we did not identify a phylogenetic clustering of either MPEC or environmental *E. coli*. There were no common patterns of STs associated with clinical mastitis, and the COGs of MPEC and the environmental *E. coli* genomes were not significantly different. In addition, the comparative genomic approach did not identify any unique virulence genes that could be attributed to MPEC to define an MPEC pathotype. However, the *fec* operon genes were significantly more common genes in MPEC genomes than in the genomes of environmental *E. coli* isolated from dairy farms. This result strongly implies that an MPEC pathotype does not exist, but rather that MPEC is an ecotype; some genes offer certain *E. coli* lineages a competitive advantage in the bovine mammary gland, eventually leading to extensive growth and inflammation.

The most abundant ST in our cattle-associated *E. coli* was ST10 (*n*=36). The association of ST10 with cattle has been reported before and demonstrates the broader applicability of this dataset and work. The ST10 is known to be one of the STs responsible for ExPEC infection in humans, and clonal complex (CC) 10, including ST10, is highly prevalent in several environments including livestock, plant-based foods, freshwater and wastewater [58,63]. The broad environmental range of CC10 may be affected by mobile genetic elements that can harbour a variety of antimicrobial resistance and virulence genes [59]. The IncF plasmid type might contribute to the adaptability of *E. coli* in the CC10 lineage. A previous study that characterized 248 CC10 *E. coli* isolates from livestock, soil, wastewater and humans found IncF was the most abundant plasmid type, containing FII (*n*=163, 66 %), FIB (*n*=122, 49%) and a wide variety of antimicrobial resistance and virulence-associated genes [59]. Our study also identified that IncF-type replicons were the most abundant plasmid replicon types in both MPEC and environmental *E. coli* groups.

*E. coli* ST58 was also common in our bovine-associated *E. coli* and is also widely distributed in food-producing animals (including cattle, poultry and swine). This sequence type is also commonly found in humans as the causative agent of ExPEC infections [64,67]. Our study identified 16 ST58 isolates from both cases of MPEC and environmental sources. Earlier work reported that ST58 isolates from cattle lack the ColV plasmid, although the ColV plasmid is common in isolates from human, porcine and avian sources [64]. In agreement with this previous work, each of the ST58 isolates from our study did not contain the ColV plasmid replicon.

The finding that the *fec* operon is important for MPEC is supported by existing literature. Gram-negative coliforms known to cause mastitis, such as *E. coli* and *Klebsiella pneumoniae*, require iron to fulfil normal metabolic processes; and iron uptake systems are known to be important in bacterial pathogenesis in general [68,69]. Bovine milk contains several iron-chelating agents including citrate, lactoferrin, transferrin, xanthine oxidase and some caseins [70]. Citrate is the principal iron-chelating agent in bovine milk [70,71]. A decrease in iron availability in this environment reduces the ability of most bacteria to grow. The *fec* iron uptake system can bind and translocate citrate-bound iron to provide a source of iron for bacteria in milk. In our study, operon genes were only commonly present in MPEC. Within the Fec system, FecA binds to the ferric citrate in the environment and passes the iron to FecB. FecC, D and E allow the iron to move across the cytoplasmic membrane. Expression of the system is regulated by sigma factor FecI and a response regulator FecR [72]. The Fec system is likely critical for MPEC to survive in the bovine udder because of the high concentration of citrate [73].

In earlier work, Leimbach *et al*. examined eight MPEC and six bovine faecal isolates and identified the *fec* operon as being enriched in MPEC isolates but also determined it was present in ~50% of faecal isolates [17]. Leimbach *et al*. also found that the fimbrial operon of the P adhesin family (*pixGFJDCHAB*), a phosphoglycerate transport operon (*pgtABCP*), the 9-O-acetyl-N-acetylneuraminic acid utilization operon (*nanSMC*) and the type 1 fimbriae operon (*fimBEAICDFGH*) were enriched in MPEC isolates [17]. By comparison, we found *fimD* (*n*=108) and *fimH* (*n*=110) in the majority of our MPEC, but the other genes were not part of the MPEC core or soft-core MPEC pan-genome. Other virulence factors commonly associated with ExPEC, such as haemolysin E (*hlyE;* 112/113 MPEC and 100/100 environmental *E. coli*) and PAI determinant (*malX;* 113/113 MPEC and 100/100 environmental *E. coli*), were commonly found in both MPEC and bovine-associated environmental *E. coli*.

A study that examined five MPEC strains in comparison to one environmental isolate also identified Fec as being present in the MPEC but absent in the environmental isolate [20]. A study that examined 66 MPEC isolates identified 19 genes on 3 loci that were correlated to MPEC, including the *fec* operon [19]. Finally, a study that conducted a bovine mastitis challenge study showed that three *E. coli* encoding Fec were able to cause a bovine mastitis infection, while a single isolate of *E. coli* without Fec was not [18]. While each of these studies suffered from low sample sizes of either MPEC or environmental comparators, our study reached similar conclusions. Given the role and presence of the *fec* operon in MPEC as core and soft-core genes, it is likely critical for MPEC to survive in the bovine udder.

The common presence of this iron uptake system in MPEC from this study and other previous studies that also characterized MPEC genomes indicates this type of *E. coli* as an ecotype rather than a pathotype. Having the Fec system allows *E. coli* to persist and grow in the iron-limiting conditions of the mammary gland. This aligns with other studies that identified the metal acquisition systems, including iron uptake systems, that enhance its fitness in the host environment, while the environmental strains lack [27,74]. For example, citrate is highly abundant in the urinary tract, and while some of it is bound to iron in this environment, the Fec system in UPEC allows this pathotype to acquire iron from this chelator and enhance their fitness in this specific environment [75]. The MPEC isolates from this study also contain several of the major virulence factors from UPEC, including *papC* (MPEC: 109/113; environmental *E. coli*: 95/100) and *fimH* (MPEC: 110/113; environmental *E. coli*: 98/100). It would be interesting to understand if there is an overlap in infectivity between MPEC and UPEC.

In this study, 113 clinical mastitis-related MPEC genomes were characterized, and a comparative genomics approach was used to identify genes that might be common to MPEC but not environmental *E. coli*. The *fec* operon genes were common in MPEC but uncommon in environmental *E. coli*. Given the agreement from our quantitative genomic analysis and the previous comparative genomic studies, the *fec* operon may be a good marker of *E. coli* strains capable of causing mastitis. In the future, the *fec* operon may be a good target for the development of novel diagnostics and therapeutics for MPEC.

## Supplementary material

10.1099/mgen.0.001436 Uncited Table S1.

10.1099/mgen.0.001436 Uncited Table S2.

10.1099/mgen.0.001436 Uncited Table S3.

10.1099/mgen.0.001436 Uncited Supplementary Material 1.

## Abbreviations

CC: clonal complex
cgSNPs: core genome single nucleotide polymorphisms
COGs: clustering orthologous groups
ExPEC: extraintestinal pathogenic *Escherichia coli*
GI: gastrointestinal
GIs: genomic islands
iTOL: Interactive Tree Of Life
MPEC: mammary pathogenic *Escherichia coli*
PAIs: pathogenicity islands
ST: sequence types
UPEC: uropathogenic *Escherichia coli*

## References

[1] Gomes F, Henriques M (2016). Control of bovine mastitis: old and recent therapeutic approaches. Curr Microbiol.

[2] Aghamohammadi M, Haine D, Kelton DF, Barkema HW, Hogeveen H (2018). Herd-level mastitis-associated costs on Canadian dairy farms. Front Vet Sci.

[3] Donovan DM, Kerr DE, Wall RJ (2005). Engineering disease resistant cattle. Transgenic Res.

[4] Bradley AJ (2002). Bovine mastitis: an evolving disease. Vet J.

[5] Klaas IC, Zadoks RN (2018). An update on environmental mastitis: challenging perceptions. Transbound Emerg Dis.

[6] Levison LJ, Miller-Cushon EK, Tucker AL, Bergeron R, Leslie KE (2016). Incidence rate of pathogen-specific clinical mastitis on conventional and organic Canadian dairy farms. J Dairy Sci.

[7] Thompson-Crispi KA, Miglior F, Mallard BA (2013). Incidence rates of clinical mastitis among Canadian Holsteins classified as high, average, or low immune responders. Clin Vaccine Immunol.

[8] Gritsenko VA, Bukharin OV (2000). The ecological and medical aspects of the symbiosis between *Escherichia coli* and man. Zh Mikrobiol Epidemiol Immunobiol.

[9] Shpigel NY, Elazar S, Rosenshine I (2008). Mammary pathogenic *Escherichia coli*. Curr Opin Microbiol.

[10] Marrs CF, Zhang L, Foxman B (2005). *Escherichia coli* mediated urinary tract infections: are there distinct uropathogenic *E. coli* (UPEC) pathotypes?. FEMS Microbiol.

[11] Foxman B, Barlow R, D’Arcy H, Gillespie B, Sobel JD (2000). Urinary tract infection: self-reported incidence and associated costs. Ann Epidemiol.

[12] Wiles TJ, Kulesus RR, Mulvey MA (2008). Origins and virulence mechanisms of uropathogenic *Escherichia coli*. Exp Mol Pathol.

[13] Karami N, Wold AE, Adlerberth I (2017). Antibiotic resistance is linked to carriage of papC and iutA virulence genes and phylogenetic group D background in commensal and uropathogenic *Escherichia coli* from infants and young children. Eur J Clin Microbiol Infect Dis.

[14] Ostblom A, Adlerberth I, Wold AE, Nowrouzian FL (2011). Pathogenicity island markers, virulence determinants malX and usp, and the capacity of *Escherichia coli* to persist in infants’ commensal microbiotas. Appl Environ Microbiol.

[15] Reidl J, Boos W (1991). The malX malY operon of *Escherichia coli* encodes a novel enzyme II of the phosphotransferase system recognizing glucose and maltose and an enzyme abolishing the endogenous induction of the maltose system. J Bacteriol.

[16] Yazdanpour Z, Tadjrobehkar O, Shahkhah M (2020). Significant association between genes encoding virulence factors with antibiotic resistance and phylogenetic groups in community acquired uropathogenic *Escherichia coli* isolates. BMC Microbiol.

[17] Leimbach A, Poehlein A, Vollmers J, Görlich D, Daniel R (2017). No evidence for a bovine mastitis *Escherichia coli* pathotype. BMC Genomics.

[18] Blum SE, Heller ED, Sela S, Elad D, Edery N (2015). Genomic and phenomic study of mammary pathogenic *Escherichia coli*. PLoS One.

[19] Goldstone RJ, Harris S, Smith DGE (2016). Genomic content typifying a prevalent clade of bovine mastitis-associated *Escherichia coli*. *Sci Rep*.

[20] Kempf F, Slugocki C, Blum SE, Leitner G, Germon P (2016). Genomic comparative study of bovine mastitis *Escherichia coli*. PLoS One.

[21] Richards VP, Lefébure T, Pavinski Bitar PD, Dogan B, Simpson KW (2015). Genome based phylogeny and comparative genomic analysis of intra-mammary pathogenic *Escherichia coli*. PLoS One.

[22] Wenz JR, Barrington GM, Garry FB, Ellis RP, Magnuson RJ (2006). *Escherichia coli* isolates’ serotypes, genotypes, and virulence genes and clinical coliform mastitis severity. J Dairy Sci.

[23] Burvenich C, Van Merris V, Mehrzad J, Diez-Fraile A, Duchateau L (2003). Severity of *E. coli* mastitis is mainly determined by cow factors. Vet Res.

[24] Blum SE, Heller ED, Jacoby S, Krifucks O, Leitner G (2017). Comparison of the immune responses associated with experimental bovine mastitis caused by different strains of *Escherichia coli*. J Dairy Res.

[25] Blum SE, Goldstone RJ, Connolly JPR, Répérant-Ferter M, Germon P (2018). Postgenomics characterization of an essential genetic determinant of mammary pathogenic *Escherichia coli*. mBio.

[26] Olson MA, Siebach TW, Griffitts JS, Wilson E, Erickson DL (2018). Genome-wide identification of fitness factors in mastitis-associated *Escherichia coli*. Appl Environ Microbiol.

[27] Yu D, Stothard P, Neumann NF (2024). Emergence of potentially disinfection-resistant, naturalized *Escherichia coli* populations across food- and water-associated engineered environments. Sci Rep.

[28] Dufour S, Labrie J, Jacques M (2019). The mastitis pathogens culture collection. Microbiol Resour Announc.

[29] Fairbrother J-H, Dufour S, Fairbrother JM, Francoz D, Nadeau É (2015). Characterization of persistent and transient *Escherichia coli* isolates recovered from clinical mastitis episodes in dairy cows. Vet Microbiol.

[30] Sears PM, McCarthy KK (2003). Diagnosis of mastitis for therapy decisions. Vet Clin North Am Food Anim Pract.

[31] Arimizu Y, Kirino Y, Sato MP, Uno K, Sato T (2019). Large-scale genome analysis of bovine commensal *Escherichia coli* reveals that bovine-adapted *E. coli* lineages are serving as evolutionary sources of the emergence of human intestinal pathogenic strains. Genome Res.

[32] Madoshi BP, Kudirkiene E, Mtambo MMA, Muhairwa AP, Lupindu AM (2016). Characterisation of commensal *Escherichia coli* isolated from apparently healthy cattle and their attendants in Tanzania. PLoS One.

[33] Bushnell B (2014). BBTools software package. https://sourceforge.net/projects/bbmap/.

[34] Souvorov A, Agarwala R, Lipman DJ (2018). SKESA: strategic k-mer extension for scrupulous assemblies. Genome Biol.

[35] Walker BJ, Abeel T, Shea T, Priest M, Abouelliel A (2014). Pilon: an integrated tool for comprehensive microbial variant detection and genome assembly improvement. PLoS One.

[36] Okonechnikov K, Conesa A, García-Alcalde F (2016). Qualimap 2: advanced multi-sample quality control for high-throughput sequencing data. Bioinformatics.

[37] Seemann T (2014). Prokka: rapid prokaryotic genome annotation. Bioinformatics.

[38] Siguier P, Perochon J, Lestrade L, Mahillon J, Chandler M (2006). ISfinder: the reference centre for bacterial insertion sequences. Nucleic Acids Res.

[39] UniProt Consortium (2019). UniProt: a worldwide hub of protein knowledge. Nucleic Acids Res.

[40] Eddy SR (2011). Accelerated profile HMM searches. PLoS Comput Biol.

[41] Haft DH, Selengut JD, Richter RA, Harkins D, Basu MK (2013). TIGRFAMs and genome properties in 2013. Nucleic Acids Res.

[42] Punta M, Coggill PC, Eberhardt RY, Mistry J, Tate J (2012). The Pfam protein families database. Nucleic Acids Res.

[43] Page AJ, Cummins CA, Hunt M, Wong VK, Reuter S (2015). Roary: rapid large-scale prokaryote pan genome analysis. Bioinformatics.

[44] Huerta-Cepas J, Forslund K, Coelho LP, Szklarczyk D, Jensen LJ (2017). Fast genome-wide functional annotation through orthology assignment by eggNOG-mapper. Mol Biol Evol.

[45] Huerta-Cepas J, Szklarczyk D, Heller D, Hernández-Plaza A, Forslund SK (2019). eggNOG 5.0: a hierarchical, functionally and phylogenetically annotated orthology resource based on 5090 organisms and 2502 viruses. Nucleic Acids Res.

[46] Kalyaanamoorthy S, Minh BQ, Wong TKF, von Haeseler A, Jermiin LS (2017). ModelFinder: fast model selection for accurate phylogenetic estimates. Nat Methods.

[47] Nguyen L-T, Schmidt HA, von Haeseler A, Minh BQ (2015). IQ-TREE: a fast and effective stochastic algorithm for estimating maximum-likelihood phylogenies. Mol Biol Evol.

[48] Letunic I, Bork P (2019). Interactive Tree Of Life (iTOL) v4: recent updates and new developments. Nucleic Acids Res.

[49] Oliveros JC (2015). Venny. An interactive tool for comparing lists with Venn’s diagrams. https://bioinfogp.cnb.csic.es/tools/venny/index.html.

[50] Brynildsrud O, Bohlin J, Scheffer L, Eldholm V (2016). Rapid scoring of genes in microbial pan-genome-wide association studies with Scoary. Genome Biol.

[51] Camacho C, Coulouris G, Avagyan V, Ma N, Papadopoulos J (2009). BLAST+: architecture and applications. BMC Bioinform.

[52] Jolley KA, Maiden MCJ (2010). BIGSdb: scalable analysis of bacterial genome variation at the population level. BMC Bioinform.

[53] Ingle DJ, Valcanis M, Kuzevski A, Tauschek M, Inouye M (2016). *In silico* serotyping of *E. coli* from short read data identifies limited novel O-loci but extensive diversity of O:H serotype combinations within and between pathogenic lineages. Microbial Genomics.

[54] Carattoli A, Zankari E, García-Fernández A, Voldby Larsen M, Lund O (2014). *In silico* detection and typing of plasmids using PlasmidFinder and plasmid multilocus sequence typing. Antimicrob Agents Chemother.

[55] Bertelli C, Laird MR, Williams KP, Lau BY, Simon Fraser University Research Computing Group (2017). IslandViewer 4: expanded prediction of genomic islands for larger-scale datasets. Nucleic Acids Res.

[56] Segura A, Auffret P, Klopp C, Bertin Y, Forano E (2017). Draft genome sequence and characterization of commensal *Escherichia coli* strain BG1 isolated from bovine gastro-intestinal tract. Stand Genomic Sci.

[57] Sartori L, Fernandes MR, Ienne S, de Souza TA, Gregory L (2017). Draft genome sequences of two fluoroquinolone-resistant CTX-M-15-producing *Escherichia coli* ST90 (ST23 complex) isolated from a calf and a dairy cow in South America. J Glob Antimicrob Resist.

[58] Manges AR, Geum HM, Guo A, Edens TJ, Fibke CD (2019). Global extraintestinal pathogenic *Escherichia coli* (ExPEC) lineages. Clin Microbiol Rev.

[59] Reid CJ, DeMaere MZ, Djordjevic SP (2019). Australian porcine clonal complex 10 (CC10) *Escherichia coli* belong to multiple sublineages of a highly diverse global CC10 phylogeny. Microb Genom.

[60] Manges AR, Harel J, Masson L, Edens TJ, Portt A (2015). Multilocus sequence typing and virulence gene profiles associated with *Escherichia coli* from human and animal sources. Foodborne Pathog Dis.

[61] Varela AR, Manageiro V, Ferreira E, Guimarães MA, da Costa PM (2015). Molecular evidence of the close relatedness of clinical, gull and wastewater isolates of quinolone-resistant *Escherichia coli*. J Glob Antimicrob Resist.

[62] Chen P-A, Hung C-H, Huang P-C, Chen J-R, Huang I-F (2016). Characteristics of CTX-M extended-spectrum β-lactamase-producing *Escherichia coli* strains isolated from multiple rivers in southern Taiwan. Appl Environ Microbiol.

[63] García V, García-Meniño I, Mora A, Flament-Simon SC, Díaz-Jiménez D (2018). Co-occurrence of mcr-1, mcr-4 and mcr-5 genes in multidrug-resistant ST10 enterotoxigenic and Shiga toxin-producing *Escherichia coli* in Spain (2006–2017). Int J Antimicrob Agents.

[64] Reid CJ, Cummins ML, Börjesson S, Brouwer MSM, Hasman H (2022). A role for ColV plasmids in the evolution of pathogenic *Escherichia coli* ST58. Nat Commun.

[65] Nüesch-Inderbinen M, Käppeli N, Morach M, Eicher C, Corti S (2019). Molecular types, virulence profiles and antimicrobial resistance of *Escherichia coli* causing bovine mastitis. Vet Rec Open.

[66] Ali A, Ali Q, Ali R, Mohsin M (2020). Draft genome sequence of an extended-spectrum β-lactamase-producing *Escherichia coli* ST58 isolate from cattle in Pakistan. J Glob Antimicrob Resist.

[67] Song J, Oh S-S, Kim J, Park S, Shin J (2020). Clinically relevant extended-spectrum β-lactamase–producing *Escherichia coli* isolates from food animals in South Korea. Front Microbiol.

[68] Lin J, Hogan JS, Smith KL (1999). Antigenic homology of the inducible ferric citrate receptor (FecA) of coliform bacteria isolated from herds with naturally occurring bovine intramammary infections. Clin Diagn Lab Immunol.

[69] Payne SM (1993). Iron acquisition in microbial pathogenesis. Trends Microbiol.

[70] Lönnerdal B, Keen CL, Hurley LS (1981). Iron, copper, zinc, and manganese in milk. Annu Rev Nutr.

[71] Hyvönen P, Haarahiltunen T, Lehtolainen T, Heikkinen J, Isomäki R (2010). Concentrations of bovine lactoferrin and citrate in milk during experimental endotoxin mastitis in early- versus late-lactating dairy cows. J Dairy Res.

[72] Braun V, Mahren S (2005). Transmembrane transcriptional control (surface signalling) of the *Escherichia coli* Fec type. FEMS Microbiol Rev.

[73] Braun V (1997). Surface signaling: novel transcription initiation mechanism starting from the cell surface. Arch Microbiol.

[74] Garcia EC, Brumbaugh AR, Mobley HLT (2011). Redundancy and specificity of *Escherichia coli* iron acquisition systems during urinary tract infection. Infect Immun.

[75] Frick-Cheng AE, Sintsova A, Smith SN, Pirani A, Snitkin ES (2022). Ferric citrate uptake is a virulence factor in uropathogenic *Escherichia coli*. mBio.

