# Supplementary material for: Comparative genomic analysis of Escherichia coli isolated from cases of bovine clinical mastitis and the dairy farm environment
Source: Microb Genom. 2025 Jun 24;11(6):001436. doi: 10.1099/mgen.0.001436 (PMC12211228; doi:10.1099/mgen.0.001436)
Supplement: Uncited Supplementary Material 1. [file mgen-11-01436-s004.pdf]

Supplementary Table 4. Prevalence of plasmids in MPEC and environmental *E. coli* genomes

Types of plasmid	Number of plasmids (n)	
	MPEC	Environmental *E. coli*
Col(MG828)_1	1	4
Col156_1	3	8
Col440I_1	5	3
Col440II_1	1	0
ColpVC_1	5	1
ColRNAI_1	0	2
IncA/C2_1	1	0
IncB/O/K/Z_1	0	1
IncB/O/K/Z_2	1	5
IncB/O/K/Z_4	0	1
IncFIA(HI1)_1_HI1	12	3
IncFIA_1	27	27
IncFIB(AP001918)_1	64	69
IncFIB(K)_1_Kpn3	7	1
IncFIB(pB171)_1_pB171	8	4
IncFIC(FII)_1	35	21
IncFII(pAMA1167-NDM-5)_1_pAMA1167-NDM-5	0	1
IncFII(pHN7A8)_1_pHN7A8	2	1
IncFII(pSE11)_1_pSE11	0	2
IncFII_1	7	5
IncHI2_1	1	10
IncHI2A_1	1	10
IncI_Gamma_1	3	4
IncI1_1_Alpha	9	10
IncI2_1	0	3
IncI2_1_Delta	3	3
IncX1_1	9	2
IncX1_4	3	1
IncX4_1	0	1
IncX4_2	1	1
IncX9_1	0	1
IncY_1	9	7
p0111_1	1	2
Total	219	214

Supplementary Table 5. IncF replicon sequence typing of MPEC and environmental *E. coli*

Strain	Plasmids	IncF RST							
		FIA	FIB	FIC	FII	FIIK	FIIS	FIIY	Sequence type
MPEC									
11800057	IncFIB(AP001918)_1, IncFII_1, IncI_Gamma_1		1		2				[F2:A-:B1]
30300071	IncFIB(AP001918)_1	6	1		24				[F24:A6:B1]
31801812	IncFIB(AP001918)_1, IncFII_1, IncI1_1_Alpha		1		2				[F2:A-:B1]
32708899	IncFIB(AP001918)_1, IncFII_1		1		2				[F2:A-:B1]
41602577	IncFIB(AP001918)_1, IncFII_1		1		2				[F2:A-:B1]
20108939	IncFIA_1, IncFIB(AP001918)_1, IncFII_1, IncI1_1_Alpha	6	10		2				[F2:A6:B10]
20309619	IncFIA_1, IncFIB(AP001918)_1	6	10		56				[F56:A6:B10]
20309640	IncFIA_1, IncFIB(AP001918)_1	6	10		56				[F56:A6:B10]
20314330	IncFIA_1, IncFIB(AP001918)_1	6	10		56				[F56:A6:B10]
10109199	IncFIB(AP001918)_1, IncFIC(FII)_1		16	1	89				[F89:A-:B16]
10216675	IncFIA_1, IncFIB(AP001918)_1, IncFIC(FII)_1	6	16	1	89				[F89:A6:B16]
10709818	IncFIB(AP001918)_1, IncFIC(FII)_1, p0111_1		16	1	89				[F89:A-:B16]
11211990	IncFIA_1, IncFIB(AP001918)_1, IncFIC(FII)_1	6	16	1	89				[F89:A6:B16]
21415616	IncFIA_1, IncFIB(AP001918)_1, IncFIC(FII)_1	6	16	1	89				[F89:A6:B16]
30415454	IncFIA_1, IncFIB(AP001918)_1, IncFIC(FII)_1	6	16	1	89				[F89:A6:B16]
32400021	IncFIB(AP001918)_1, IncFIC(FII)_1, IncI1_1_Alpha		16	1	89				[F89:A-:B16]

40115481	IncFIA_1, IncFIB(AP001918)_1, IncFIC(FII)_1	6	16	1	89				[F89:A6:B16]
41100011	IncFIA_1, IncFIB(AP001918)_1	6	16		56				[F56:A6:B16]
41505922	IncFIB(AP001918)_1, IncFIC(FII)_1		16	1	89				[F89:A-:B16]
20202040	IncFIA_1, IncFIB(AP001918)_1	6	20		22*				[F-:A6:B20]
21014604	IncFIA_1, IncFIB(AP001918)_1	6	20		22*				[F-:A6:B20]
10407462	IncFIB(pB171)_1_pB171, IncY_1		23		19*				[F-:A-:B23]
10816417	IncFIB(pB171)_1_pB171, IncY_1		23		19*				[F-:A-:B23]
20814168	IncFIB(pB171)_1_pB171		23		19*				[F-:A-:B23]
21314933	IncFIA_1, IncFIB(pB171)_1_pB171	6*	23		57				[F57:A-:B23]
21914232	IncFIA_1, IncFIB(pB171)_1_pB171	2	23		19*				[F-:A2:B23]
22113962	IncFIB(pB171)_1_pB171, IncX1_1		23		19*				[F-:A-:B23]
40816739	IncFIA(HI1)_1_HI1, IncFIB(pB171)_1_pB171	19*	23		57				[F57:A-:B23]
41809617	IncFIA(HI1)_1_HI1, IncFIB(pB171)_1_pB171	19*	23		57				[F57:A-:B23]
20510930	IncFIA(HI1)_1_HI1, IncFIB(AP001918)_1, IncFIC(FII)_1	19	24	5*	75				[F75:A19:B24]
41810132	IncFIA(HI1)_1_HI1, IncFIB(AP001918)_1, IncFIC(FII)_1, IncI_Gamma_1, IncX1_1	19	24	5*	75				[F75:A19:B24]
10800294	IncFIB(AP001918)_1		38		24				[F24:A-:B38]
40202761	IncB/O/K/Z_2, IncFIA_1, IncFIB(AP001918)_1	6	38		67				[F67:A6:B38]
41613979	IncFIA_1, IncFIB(AP001918)_1	6	38		67				[F67:A6:B38]

10700396	IncFIB(AP001918)_1, IncI1_1_Alpha, IncI2_1_Delta, IncX4_2		41		74				[F74:A-:B41]
21413445	IncFIB(AP001918)_1		42		112				[F112:A-:B42]
21317521	IncFIA_1, IncFIB(AP001918)_1, IncFIC(FII)_1	6	43	1	89				[F89:A6:B43]
21317859	IncFIA_1, IncFIB(AP001918)_1	6*	48		52				[F52:A-:B48]
21012914	IncFIB(AP001918)_1		67		68*				[F-:A-:B67]
21202100	IncFIA_1, IncFIB(AP001918)_1	2	16*		56				[F56:A2:B-]
22114969	IncFIA_1, IncFIB(AP001918)_1	2	16*		56				[F56:A2:B-]
22406217	IncFIA_1, IncFIB(AP001918)_1	2	16*		56				[F56:A2:B-]
31716253	IncFIA(HI1)_1_HI1, IncFIB(K)_1_Kpn3		18*						[F-:A-:B-]
31716895	IncFIA(HI1)_1_HI1, IncFIB(K)_1_Kpn3		18*						[F-:A-:B-]
10715833	IncFIA_1, IncFIB(AP001918)_1, IncI2_1_Delta	6	24*		56				[F56:A6:B-]
22710987	Col156_1, IncFIB(AP001918)_1, IncY_1		31*		52				[F52:A-:B-]
22115768	IncFIB(AP001918)_1, IncFIC(FII)_1		42*	1	89				[F89:A-:B-]
41207659	IncFIA_1, IncFIB(AP001918)_1, IncI_Gamma_1, IncX1_1	6	48*		104, 13				[F104:A6:B-]
41300398	IncFIA_1, IncFIB(AP001918)_1, IncFII_1	6	59*		2				[F2:A6:B-]
10208472	IncFIB(AP001918)_1, IncFIC(FII)_1		67*		64*				[F-:A-:B-]
10209356	IncFIB(AP001918)_1, IncFIC(FII)_1		67*		64*				[F-:A-:B-]
10213407	IncFIB(AP001918)_1, IncFIC(FII)_1		67*		64*				[F-:A-:B-]
10415566	IncFIB(AP001918)_1, IncFIC(FII)_1		67*		64*				[F-:A-:B-]
10417409	IncFIC(FII)_1		67*		64*				[F-:A-:B-]

11603481	IncFIB(AP001918)_1, IncFIC(FII)_1		67*		64*				[F-:A-:B-]
20508456	IncFIB(AP001918)_1, IncFIC(FII)_1		67*		64*				[F-:A-:B-]
21210525	IncFIB(AP001918)_1, IncFIC(FII)_1		67*		64*				[F-:A-:B-]
21212574	IncFIB(AP001918)_1, IncFIC(FII)_1		67*		64*				[F-:A-:B-]
22510686	IncFIB(AP001918)_1, IncFIC(FII)_1		67*		64*				[F-:A-:B-]
30215009	IncFIB(AP001918)_1, IncFIC(FII)_1		67*		64*				[F-:A-:B-]
30810358	IncFIB(AP001918)_1, IncFIC(FII)_1		67*		64*				[F-:A-:B-]
30812765	IncFIB(AP001918)_1, IncFIC(FII)_1		67*		64*				[F-:A-:B-]
31209373	IncFIB(AP001918)_1, IncFIC(FII)_1, IncFII(pHN7A8)_1_pHN7A8, IncI1_1_Alpha		67*		40				[F40:A-:B-]
31412124	IncFIB(AP001918)_1, IncFIC(FII)_1		67*		64*				[F-:A-:B-]
31501194	IncFIB(AP001918)_1, IncFIC(FII)_1		67*		64*				[F-:A-:B-]
31709637	IncFIB(AP001918)_1, IncFIC(FII)_1		67*		64*				[F-:A-:B-]
32608571	IncFIB(AP001918)_1, IncFIC(FII)_1		67*		64*				[F-:A-:B-]
40317434	IncFIB(AP001918)_1, IncFIC(FII)_1		67*		64*				[F-:A-:B-]
40614038	IncFIB(AP001918)_1, IncFIC(FII)_1, IncFII(pHN7A8)_1_pHN7A8		67*		40				[F40:A-:B-]
40717203	IncFIB(AP001918)_1, IncFIC(FII)_1		67*		64*				[F-:A-:B-]
41701140	IncFIB(AP001918)_1, IncFIC(FII)_1		67*		64*				[F-:A-:B-]
11909132	IncFIA(HI1)_1_HI1, IncFIB(K)_1_Kpn3	18*							[F-:A-:B-]
21013690	IncFIA(HI1)_1_HI1, IncFIB(K)_1_Kpn3	18*							[F-:A-:B-]
21310034	IncFIA_1, IncFIB(pB171)_1_pB171								[F-:A-:B-]

21914256	IncFIA(HI1)_1_HI1, IncFIB(K)_1_Kpn3	18*							[F-:A-:B-]
31313964	IncFII_1				106*				[F-:A-:B-]
32608632	Col440I_1, IncFIA(HI1)_1_HI1, IncFIB(K)_1_Kpn3	18*							[F-:A-:B-]
40611716	IncFIA(HI1)_1_HI1, IncY_1	18*							[F-:A-:B-]
40714004	IncFIA(HI1)_1_HI1, IncFIB(K)_1_Kpn3	18*							[F-:A-:B-]
Commensal bovine *E. coli*									
BM117	IncFIB(AP001918)_1, IncFII_1		1		2				[F2:A-:B1]
BM321	IncFIB(AP001918)_1		1		24				[F24:A-:B1]
RiKo2299-09	IncFIA_1, IncFIB(AP001918)_1, Col156_1	4	1		36				[F36:A4:B1]
RiKo2308-09	IncB/O/K/Z_2, IncFIA_1, IncFIB(AP001918)_1, Col156_1	4	1		36				[F36:A4:B1]
RiKo2340-09	IncFIA_1, IncFIB(AP001918)_1, Col156_1	4	1		36				[F36:A4:B1]
VL49	IncFIB(AP001918)_1, IncFII_1		1		2				[F2:A-:B1]
35-1BK	IncFIB(AP001918)_1		3		23				[F23:A-:B3]
57-K120-A	IncFIA_1, IncFIB(AP001918)_1	6	10		56				[F56:A6:B10]
58-K131-A	IncI_Gamma_1, IncFIB(AP001918)_1		10						[F-:A-:B10]
65-L97-A	IncFIB(AP001918)_1, IncFIC(FII)_1		10	1	89				[F89:A-:B10]
BE2534	IncFIA_1, IncFIB(AP001918)_1	6	10		56				[F56:A6:B10]
13-1BK	Col156_1, IncFIB(AP001918)_1, p0111_1		15						[F-:A-:B15]
36-1BK	IncHI2_1, IncHI2A_1, IncFIB(AP001918)_1, IncI1_1_Alpha, IncFIA_1	2	16						[F-:A2:B16]
61-K142-A	IncFIA_1, IncFIB(AP001918)_1	6	16		56				[F56:A6:B16]

AF83	IncFIB(AP001918)_1		16		56				[F56:A-:B16]
AF84	IncFIB(AP001918)_1, IncFIA_1	2	16		57				[F57:A2:B16]
AF94	IncFIA_1, IncFIB(AP001918)_1, IncI1_1_Alpha	6	16		56				[F56:A6:B16]
AF97	Col440I_1, IncFIB(AP001918)_1		16						[F-:A-:B16]
BE2494	IncFIA_1, Col(MG828)_1, IncFIB(AP001918)_1, IncFIC(FII)_1	6	16	1	89				[F89:A6:B16]
BE2525	IncFIA_1, IncFIB(AP001918)_1, IncI2_1_Delta, IncFIC(FII)_1	6	16	1	89				[F89:A6:B16]
BE2774	IncFIA_1, IncFIB(AP001918)_1	6	16		112				[F112:A6:B16]
BE734	IncFIA_1, IncHI2A_1, IncFIB(AP001918)_1, IncHI2_1	6	16		112				[F112:A6:B16]
BM116	IncFIA_1, IncFIB(AP001918)_1, IncFIC(FII)_1	6	16	1	89				[F89:A6:B16]
K71	IncFIA_1, IncFIB(AP001918)_1	6	16		56				[F56:A6:B16]
KK-NP003	IncI_Gamma_1, IncFIB(AP001918)_1, IncHI2_1, IncHI2A_1		16		56				[F56:A-:B16]
RiKo2351-09	IncFIB(AP001918)_1		16		24				[F24:A-:B16]
VL122	IncFIA_1, IncI2_1, IncFIB(AP001918)_1, IncFIC(FII)_1	6	16	1	89				[F89:A6:B16]
VL128	IncFIA_1, IncFIB(AP001918)_1, IncFIC(FII)_1	6	16	1	89				[F89:A6:B16]
56-K145-A	IncFIC(FII)_1, IncFIB(AP001918)_1		20	4*	46				[F46:A-:B20]
K34	IncFIA_1, IncFIB(AP001918)_1, IncI1_1_Alpha	6	20		19*				[F-:A6:B20]
VL123	IncFIA_1, IncFIB(AP001918)_1	6	20		22*				[F-:A6:B20]

14-1BK	IncY_1, IncFIB(pB171)_1_pB171		23		104				[F104:A6:B23]
16-1BK	IncY_1, IncFIB(pB171)_1_pB171		23		104				[F104:A6:B23]
BE1806	IncFIB(pB171)_1_pB171		23		19*				[F-:A-:B23]
BM447	Col(MG828)_1, IncFIA(HI1)_1_HI1, ColRNAI_1, IncX1_4, IncFIB(pB171)_1_pB171, IncX9_1	19*	23		57				[F57:A-:B23]
16-B15-A	IncFIB(AP001918)_1		38		19*				[F-:A-:B38]
28-1BK	IncHI2A_1, IncFIB(AP001918)_1, IncHI2_1		42		104				[F104:A-:B42]
32-1BK	Col440I_1, IncHI2A_1, IncFIB(AP001918)_1, IncHI2_1		42		57				[F57:A-:B42]
5-1BK	IncHI2A_1, IncFIB(AP001918)_1, IncHI2_1		42		57				[F57:A-:B42]
AF103	IncFIB(AP001918)_1		42		22*				[F-:A-:B42]
Ec47VL	ColRNAI_1, IncFIB(AP001918)_1, IncY_1, IncFIA_1, IncFII(pAMA1167-NDM-5)_1_pAMA1167-NDM-5	1	49		1				[F1:A1:B49]
3-1BK	IncFIB(AP001918)_1		54		57				[F57:A-:B54]
BE2805	Col156_1, IncFIB(AP001918)_1		56						[F-:A-:B56]
KK-NP001	IncFIB(AP001918)_1		56						[F-:A-:B56]
AF85	IncFIB(AP001918)_1, IncFIC(FII)_1, IncI1_1_Alpha		59	1	89				[F89:A-:B59]
62-L26-A	IncFIB(AP001918)_1		61		56				[F56:A-:B61]
BM304	IncFIA_1, IncFIC(FII)_1, IncFIB(AP001918)_1	6	61		16*				[F-:A6:B61]

VL21	IncFIB(AP001918)_1		61		56				[F56:A-:B61]
HH-NP010	IncB/O/K/Z_1, IncFIB(AP001918)_1		14*						[F-:A-:B-]
20-B54-A	IncFIC(FII)_1, IncFIB(AP001918)_1		15*		10				[F10:A-:B-]
VL68	IncFIC(FII)_1, IncFIB(AP001918)_1, IncI1_1_Alpha		15*		10				[F10:A-:B-]
63-L27-A	IncFIB(AP001918)_1, IncFIC(FII)_1		2*	1	89				[F89:A6:B-]
HH-NP008	IncB/O/K/Z_2, IncFIB(AP001918)_1		20*						[F-:A-:B-]
RiKo2305-09	IncFIA_1, IncFIB(AP001918)_1	4	21*		36				[F36:A4:B-]
18-B32-A	IncFIA_1, IncFIB(AP001918)_1	6	24*		56				[F56:A6:B-]
BE2454	IncFII_1, IncFIB(AP001918)_1		42*		8*				[F-:A-:B-]
40-1BK	IncB/O/K/Z_2, IncFIC(FII)_1, IncFIB(AP001918)_1, IncI2_1_Delta, IncHI2A_1, IncFIA_1, IncHI2_1	2	43*		10				[F10:A2:B-]
45-1BK	ColpVC_1, IncFIA_1, IncFIB(AP001918)_1, IncY_1, IncFIC(FII)_1	3*	43*	1	89				[F89:A-:B-]
BE854	IncFIB(AP001918)_1		43*		57				[F57:A-:B-]
HH-P025	IncFIA_1, IncFIB(AP001918)_1	6	45*		63*				[F-:A6:B-]
BM449	IncFIB(AP001918)_1		46*		74				[F74:A-:B-]
12-1BK	IncB/O/K/Z_2, IncFIB(AP001918)_1		59*						[F-:A-:B-]
27-1BK	IncFIB(AP001918)_1, IncHI2A_1, IncHI2_1		59*		56				[F56:A-:B-]
59-K139-A	IncFIC(FII)_1, IncFIB(AP001918)_1, IncI2_1		59*		40				[F40:A-:B-]
67-L119-A	IncFIC(FII)_1, IncFIB(AP001918)_1		59*		10				[F10:A-:B-]
HH-NP006	IncI2_1_Delta, IncFIB(AP001918)_1, IncX4_2, IncI1_1_Alpha		59*						[F-:A-:B-]

HH-NP033	IncFIB(AP001918)_1, IncFIC(FII)_1		59*	1					[C1:A-:B-]
VL119	IncFIC(FII)_1, IncFIB(AP001918)_1, IncI1_1_Alpha		59*						[F-:A-:B-]
VL66a	IncFIC(FII)_1, IncFIB(AP001918)_1, IncI1_1_Alpha		59*		10				[F10:A-:B-]
19-B39-A	IncFIC(FII)_1, IncFII(pHN7A8)_1_pHN7A8		67*		40				[F40:A-:B-]
66-L112-A	IncFIC(FII)_1, IncFIB(AP001918)_1		67*		64*				[F-:A-:B-]
AF90	IncFIB(AP001918)_1, IncI1_1_Alpha		67*		22*				[F-:A-:B-]
6-1BK	IncFII_1, IncY_1, IncFIA_1	2			64*				[F-:A2:B-]
HH-P022	Col(MG828)_1, Col156_1, IncFII(pSE11)_1_pSE11				79*				[F-:A-:B-]
KK-NP002	IncI_Gamma_1, IncFIA(HI1)_1_HI1, IncFIB(K)_1_Kpn3, IncFII_1	18			104, 2				[F104:A18:B-]
VL115	IncFIA(HI1)_1_HI1, IncX1_1, IncFII(pSE11)_1_pSE11				79*				[F-:A-:B-]

*: Novel allele, ST may indicate nearest ST (identity >97%, 100% of coverage)
